# Transcatheter Aortic Valve Replacement in Patients Aged 65 Years and Younger: Unresolved Issues and Future Directions

**DOI:** 10.3390/life16071075

**Published:** 2026-06-27

**Authors:** Julius Jelisejevas, Giacomo Maria Cioffi, Ioannis Skalidis, Serban Puricel, Ali Husain, David A. Wood, Mariama Akodad, Peter Wenaweser, Pascal Meier, Mario Togni, Stéphane Cook

**Affiliations:** 1 Department of Cardiology, University and Hospital Fribourg, Chemin des Pensionnats 2-6, 1708 Fribourg, Switzerland; 2 Sheikh Jaber Al-Ahmad Hospital, Kuwait City 10002, Kuwait; 3 Dilawri Cardiovascular Institute, Vancouver, BC V6Z 2W5, Canada; 4 Institut Cardiovasculaire Paris-Sud, Hôpital Jacques Cartier, 91300 Massy, France

**Keywords:** TAVR, TAVI, aortic stenosis, TAVR in young patients, lifetime management, TAVR in patients ≤65

## Abstract

Introduction: Transcatheter aortic valve replacement (TAVR) has become the predominant treatment for severe aortic stenosis across all surgical risk categories. However, its role in patients aged 65 years and younger remains uncertain, and current guideline recommendations continue to favor surgical aortic valve replacement (SAVR) in this population. Despite this, contemporary real-world data demonstrate a marked increase in TAVR utilization among younger patients, creating an important gap between guidelines and clinical practice. Methods: This review synthesizes contemporary observational evidence evaluating TAVR in patients ≤65 years, with a focus on patient selection, clinical outcomes, and lifetime management considerations. Results: Available studies demonstrate that younger patients undergoing TAVR often represent a highly selected and clinically complex population with greater comorbidity burden, higher surgical risk, and shorter life expectancy than age-matched SAVR recipients, yet substantial hospital-level variation in TAVR utilization exists even after risk adjustment. Mid-term observational data suggest higher mortality and heart failure readmission rates following TAVR compared with SAVR, although these findings are likely influenced by substantial baseline differences between treatment groups. No randomized controlled trial has specifically compared TAVR and SAVR in patients ≤65 years. Furthermore, long-term issues including valve durability, coronary access, redo-TAVR feasibility, and THV optimization remain incompletely understood. Conclusions: TAVR recipients ≤65 are often a clinically distinct group characterized by significantly heavier comorbidity burdens than SAVR recipients of the same age with standard surgical risk models possibly underestimating the true clinical risk. Despite this, significant hospital-level variation in TAVR utilization persists even after risk adjustment, suggesting that institutional practice patterns and other non-clinical factors continue to influence treatment selection.

## 1. Introduction

Transcatheter aortic valve replacement (TAVR) has undergone a remarkable evolution since its clinical introduction, progressing from a salvage procedure in inoperable patients to the dominant intervention of aortic valve replacement (AVR) across all surgical risk groups [1,2,3,4]. Despite this progress, the role of TAVR in patients aged 65 years and younger remains speculative. The fundamental challenge is lifelong management with younger patients expected to outlive the durability of any bioprosthetic valve, which (whether surgical or transcatheter) are subject to structural valve deterioration (SVD) over time, with durability data beyond 10 years remaining limited for either platform [5,6,7,8]. For TAVR specifically, concerns extend further to coronary access at the time of future interventions and the feasibility of redo-TAVR [9,10,11,12,13,14,15]. The latest American College of Cardiology/American Heart Association (ACC/AHA) and European Society of Cardiology/European Association for Cardio-Thoracic Surgery (ESC/EACTS) guidelines recommend SAVR over TAVR for patients younger than 65/70 years respectively, or with an expected life expectancy >20 years [16,17]. Nevertheless, real-world practice has diverged from guideline recommendations, with several large registry and database studies reporting that TAVR utilization in patients ≤65 years undergoing AVR has increased [18,19,20,21,22,23,24,25]. This guideline-practice gap raises the question: What are the key factors driving TAVR utilization in young patients? This review synthesizes contemporary observational evidence, including large registries and cohort studies, reporting outcomes of TAVR in patients ≤65 years, and examines current global trends in patient selection, outcomes, and future directions.

## 2. Methods

This article was conducted as a narrative review in accordance with recommendations for high-quality narrative reviews and the Scale for the Assessment of Narrative Review Articles (SANRA). A literature search was performed in PubMed/MEDLINE, Embase and the Cochrane Library for studies published in English between January 2010 and April 2026. A comprehensive search strategy combined controlled vocabulary and keywords related to TAVR and young patients: “transcatheter aortic valve replacement” OR “transcatheter aortic valve implantation” OR TAVR OR TAVI AND (young OR younger OR age <65 OR ≤65 OR under 70 OR low-risk OR lifetime management OR bicuspid) AND (outcomes OR mortality OR complications OR structural valve degeneration). Database searches were adapted to each platform. Registries, observational cohort studies, and database analyses that specifically reported outcomes of TAVR in patients aged ≤65 years were included. Case reports, small case series, non-English publications, and studies not specifically reporting outcomes in patients aged 65 years and younger were excluded. To maximize sample size, studies reporting outcomes in populations aged <65 years as well as those aged ≤65 years were eligible for inclusion. These cohorts were grouped and are collectively described as ≤65 years throughout the manuscript to facilitate comparison across studies and provide a consistent framework for discussion. Data were extracted on patient characteristics, patient selection, study design, follow-up duration and clinical outcomes.

### Current Guideline Recommendations for Aortic Valve Replacement

The 2020 ACC/AHA guidelines provide age- and life expectancy-based recommendations for the selection of TAVR versus SAVR [16]. For patients younger than 65 years of age or with a life expectancy exceeding 20 years, SAVR is recommended when patient characteristics are otherwise suitable. The 2020 ACC/AHA guidelines acknowledge the role of shared decision-making for younger patients, recognizing that informed patient preferences may lead to TAVR being pursued. However, the guideline is clear that TAVR should not be the default choice simply on the grounds of procedural invasiveness in this age group. The 2025 ESC/EACTS guidelines recommended SAVR for patients at low surgical risk under 70 years of age (defined as STS-PROM or EuroSCORE II <4%), with TAVR reserved for patients 70 years or older or those at high surgical risk (STS-PROM or EuroSCORE II >8%) [17]. The 2025 guidelines place heightened emphasis on a lifetime management framework, explicitly recognizing that the feasibility of future coronary access, redo-TAVR, and patient-specific anatomy must inform the choice of initial intervention. The guidelines maintain that, while age is an important factor, it should function within a broader matrix of anatomical features, concomitant disease, and estimated life expectancy rather than serving as an absolute threshold.

## 3. Real-World Experience with TAVR in Patients Aged 65 Years and Younger

### 3.1. Temporal Trends in TAVR Utilization in Patients ≤65 Years

Multiple large-scale registries and observational studies have documented a rapid and sustained increase in TAVR utilization among patients ≤65 years over the past decade, Table 1 and graphical abstract [18,19,20,21,22,23,24,25]. In the three-state cohort study by Alabbadi et al. (2025) encompassing California, New York, and New Jersey, the proportion of patients under 65 years with aortic stenosis (AS) undergoing TAVR rather than isolated bioprosthetic SAVR rose from 7.1% in 2013 to 54.7% in 2021 [23]. This trend was consistent across all three states. Similarly, data from the Nationwide Readmissions Database analyzed by Ismayl et al. (2025) demonstrated a rise from 12.6% to 41.4% in the proportion of AVR performed using TAVR among patients aged 50 to 64 years between 2016 and 2021 [22]. In the Northern New England Cardiovascular Disease Group registry (Gupta et al., 2025), TAVR utilization more than doubled across the study period (2015–2023), reaching near equalization with isolated bioprosthetic SAVR by 2021–2023 (28.3% vs. 30.8% of all AVR procedures) [24]. In Europe, the pattern was substantially more conservative. Prosperi-Porta et al. (2023) reported that TAVR represented only 11.1% of all AVR procedures in patients under 65 years by 2020, compared with the dominant TAVR utilization seen in older age groups [18]. While the absolute number of younger patients undergoing TAVR increased by 63.2% between 2015 and 2020, TAVR remained uncommon in this age group in France and was predominantly restricted to high-risk patients who were not suitable candidates for SAVR. The hospital-level analysis by Glance et al. (2026) revealed that substantial variation exists in TAVR versus SAVR utilization among patients under 65 years, even after adjustment for patient characteristics [25]. The median hospital TAVR rate was 52.9% (IQR 35.3–70.7%), and patients treated at hospitals with higher risk-adjusted TAVR rates had 2.7-fold higher odds of undergoing TAVR compared with patients at lower-utilization hospitals. Nearly half (46.5%) of the lowest-risk patients—those with a predicted surgical mortality below 0.5%—underwent TAVR.

### 3.2. Comorbidity Profile of TAVR Patients ≤65 Years: Comparison with SAVR Recipients ≤65 Years

A consistent finding across the studies is that patients ≤65 years who undergo TAVR show a substantially heavier comorbidity burden than SAVR recipients of comparable age. In the Northern New England registry (Gupta et al., 2025), TAVR patients under 65 had a significantly higher STS score (median 2.5% versus 0.9% for isolated SAVR; *p* < 0.001) and were more likely to be classified as intermediate or high surgical risk (37.3% versus 2.7% in isolated SAVR) [24]. The TAVR cohort had markedly higher rates of hypertension (80.7% versus 63.9%), diabetes (50.4% versus 28.3%), prior percutaneous coronary intervention (27.0% versus 5.4%), prior coronary artery bypass grafting (15.7% versus 1.2%), atrial fibrillation (19.7% versus 2.6%), peripheral arterial disease (25.9% versus 3.6%), and immunocompromised status (12.8% versus 4.4%) compared with isolated SAVR patients. Approximately 50% of TAVR recipients under 65 in this study were categorized as high or extreme surgical risk by the local heart team. The international multicenter study by Bække et al. (2025), which enrolled 459 TAVR recipients ≤65 from nine centers across Europe and the United States between 2010 and 2019, provides detailed insight into the reasons why SAVR was declined [19]. The predominant factors documented by heart teams were prior cardiac surgery (35%), chronic pulmonary disease (30%), frailty (23%), poor left ventricular systolic function (19%), chronic kidney disease (18%), and active malignancy or prior chest radiation (15%). Notably, approximately two-thirds of patients had more than one risk factor for SAVR denial, underscoring the multimorbid nature of this cohort. In this population, the median STS predicted mortality was 2.6% (nominally within the low-risk category by conventional thresholds), yet the clinical complexity was substantial. The single-institution study from the University of Pittsburgh Medical Center (Ashwat et al., 2025) confirmed these findings, demonstrating that even after propensity score matching, TAVR patients under 65 had significantly higher STS scores (median 3.9% versus 1.8% in matched SAVR patients) and substantially higher rates of congestive heart failure (84.5%), chronic lung disease (48.5%), and diabetes (47.4%) [21]. The three-state analysis by Alabbadi et al. (2025) similarly found that unmatched TAVR recipients were older, more likely to be frail, and carried more comorbid conditions than SAVR recipients [23]. Bicuspid aortic valves were more common in the SAVR cohort (14.8% versus 5.0% in TAVR), reflecting the anatomical considerations that typically favor a surgical approach in this subset.

### 3.3. Comorbidity Profile of TAVR Patients ≤65 Years: Comparison with TAVR Recipients Aged 65–80 Years

The large-scale analysis by Coylewright et al. (2025), using data from the STS/ACC TVT Registry on 139,695 patients who underwent balloon-expandable TAVR between 2019 and 2023, provides the most comprehensive comparative characterization of younger TAVR recipients versus their older counterparts [20]. Among 13,849 patients younger than 65 years who underwent TAVR in this analysis, the comorbidity profile was, in several key respects, more severe than that of the 125,846 patients aged 65 to 80 years who underwent the same procedure. Patients younger than 65 years were significantly more likely to have congestive heart failure within two weeks of the procedure (68.9% versus 65.3%), chronic lung disease (32.4% versus 27.1%), diabetes (47.3% versus 43.9%), immunocompromised status (9.9% versus 6.6%), end-stage kidney disease requiring dialysis (11.7% versus 4.0%), a hostile chest (4.9% versus 2.7%), and cardiogenic shock within 24 h (2.3% versus 0.8%). Younger patients also had significantly worse baseline quality of life as assessed by the Kansas City Cardiomyopathy Questionnaire (mean score 47.7 versus 52.9; *p* < 0.001). The mean STS-predicted mortality was paradoxically lower in the under-65 cohort (mean 3.1% versus 3.3%) compared with the 65–80 age group, despite the younger patients’ heavier burden of non-cardiac comorbidities. The bicuspid aortic valve prevalence was markedly higher in the under-65 cohort (25.2% versus 7.6% in those aged 65–80 years).

### 3.4. Clinical Outcomes of TAVR in Patients ≤65 Years: Short-Term and In-Hospital Outcomes

Short-term outcomes after TAVR in patients ≤65 years appear generally acceptable across the reviewed studies, though not uniformly favorable when compared with SAVR in propensity-matched analyses. Ismayl et al. (2025) reported that TAVR in patients aged 50–64 years was associated with significantly lower in-hospital mortality (1.0% versus 2.0%), lower rates of stroke (1.4% versus 2.8%), acute kidney injury (9.8% versus 17.4%), and major bleeding (1.0% versus 1.4%) compared with SAVR, along with shorter length of stay (median 2 versus 6 days) and fewer non-home discharges (17.1% versus 54.6%) [22]. The European multicenter cohort (Bække et al., 2025) reported a 30-day permanent pacemaker (PPM) implantation rate of 12%, 30-day mortality of 4%, and major bleeding or vascular complications in approximately 5% of patients each [19]. In the single-center propensity-matched analysis by Ashwat et al. (2025), there were no significant differences between matched TAVR and SAVR cohorts in most immediate postoperative complications, including operative mortality (0% versus 1.0%), stroke or TIA (0% versus 3.1%), or new-onset dialysis [21]. As expected, hospital length of stay and blood product transfusion were significantly higher in the SAVR group.

### 3.5. Clinical Outcomes of TAVR in Patients ≤65 Years: Mid-Term and Long-Term Outcomes

The mid- and long-term outcome data consistently demonstrate worse survival for TAVR compared with SAVR in patients ≤65, although the magnitude of difference and degree of certainty vary by study design and follow-up duration. The three-state registry analysis by Alabbadi et al. (2025) found that after propensity score matching of 1994 pairs, 6-year all-cause mortality was significantly higher after TAVR (23.3%) compared with isolated bioprosthetic SAVR (10.5%), corresponding to a hazard ratio of 2.27 (95% CI 1.82–2.83; *p* < 0.001) [23]. This survival disadvantage persisted across multiple sensitivity analyses, including restriction to patients aged 50–64 years, to procedures performed after the low-risk TAVR approval in 2019, to non-dialysis patients, and after overlap weighting. In the single-center UPMC study (Ashwat et al., 2025), five-year survival estimates were markedly lower in matched TAVR patients (46.3%; 95% CI 34.2–58.6%) compared with SAVR patients (79.7%; 95% CI 69.5–88.2%; log-rank *p* < 0.001) [21]. The cumulative incidence of heart failure readmission at five years was more than double in the TAVR group (43.1% versus 20.8%; *p* < 0.001). In the multinational multicenter study by Bække et al. (2025), comparing 459 TAVR recipients ≤65 years with 1836 age-, sex-, and comorbidity-matched controls from the Danish National Registries, three-year all-cause mortality was 34% in the TAVR group compared with 8% in matched controls—corresponding to a hazard ratio of 6.5 (95% CI 4.5–9.6; *p* < 0.001) [19]. Notably, one-year outcomes from the TVT Registry (Coylewright et al., 2025) showed that patients younger than 65 had a significantly higher one-year readmission rate (28.2% versus 26.1%; *p* < 0.001) and a trend toward higher all-cause mortality (9.9% versus 8.2%; *p* < 0.001) compared with patients aged 65–80 [20]. After propensity matching, readmission rates remained significantly higher in the younger cohort (28.2% versus 26.8%; *p* = 0.03), while one-year mortality was similar (9.9% versus 10.1%; *p* = 0.55).

## 4. Discussion

### 4.1. TAVR Utilization in Patients ≤65

The comorbidity data strongly suggest that many younger patients who have received TAVR in contemporary practice are poor or high-risk surgical candidates, for whom TAVR represents a clinically appropriate alternative to SAVR [19,20,21,24]. The high rates of prior cardiac surgery, dialysis, severe lung disease, frailty, and active malignancy documented in TAVR registries support the interpretation that heart team decision-making in this age group is clinically justified. On the other hand, the hospital-level variation data from Glance et al. (2026) reveal that institutional practice culture and non-clinical factors exert a substantial influence on procedure selection [25]. The fact that nearly half of the lowest-risk patients under 65 underwent TAVR rather than SAVR conflicts directly with guideline recommendations. These patients should be considered excellent SAVR candidates, but this can also depict the limits of STS scores, which might underestimate the true surgical risk. It is important to note that the comparator populations across studies are not equivalent. Patients ≤65 receiving TAVR in registries and databases are not directly comparable with SAVR recipients of the same age in terms of comorbidities and life expectancy. Propensity matching cannot fully account for the clinical judgment of the heart team regarding the overall prognosis that drives procedure selection. Any comparison of outcomes between TAVR and SAVR in this age group must therefore be interpreted with caution, recognizing that TAVR recipients systematically carry a higher burden of disease that may independently account for a substantial proportion of the observed survival differences. In other words, patients undergoing SAVR are typically characterized by a lower burden of comorbidities and a more favorable baseline risk profile, which may partly explain the observed differences in outcomes.

### 4.2. The Absence of Randomized Controlled Trial Data

There is a complete absence of randomized controlled trials (RCTs) data specifically addressing TAVR versus SAVR in patients ≤65 years of age. The low-risk RCTs enrolled predominantly older patients and excluded bicuspid aortic valve (BAV) disease; fewer than 10% of their participants were younger than 65 [3,4]. Subgroup analyses of younger patients within these trials are underpowered and do not provide reliable comparative evidence.

### 4.3. Bicuspid Aortic Valve: A Specific Consideration in Young Patients

Bicuspid aortic valve requires specific attention in the context of TAVR in young patients, given its disproportionate prevalence in this age group. In the TVT Registry analysis (Coylewright et al., 2025), 25.2% of TAVR recipients under 65 had BAV disease, compared with 7.6% of those aged 65–80. In contrast, BAV prevalence was substantially lower in TAVR recipients than in SAVR recipients in most registry studies—16.4% in the Northern New England TAVR cohort versus 47.2% in isolated SAVR patients (Gupta et al., 2025)—and only 5.0% in the TAVR group versus 14.8% in SAVR (Alabbadi et al., 2025) [20,23,24]. This reflects both the anatomical challenges of TAVR in BAV and the exclusion of BAV from pivotal low-risk TAVR trials. Although contemporary transcatheter techniques and newer-generation devices have improved outcomes in BAV patients, TAVR in bicuspid anatomy remains associated with higher rates of paravalvular regurgitation, procedural complexity and stroke if compared with tricuspid anatomy, with limited long-term follow-up data [26,27,28,29]. In patients ≤65 with BAV disease, SAVR remains the preferred approach in most cases.

### 4.4. Lifetime Management: The Pathway Consideration

One of the most consequential implications of performing TAVR in a patient ≤65 is the commitment to a specific pathway for lifetime management. Unlike a mechanical surgical valve—which carries the need for lifelong anticoagulation but offers potentially decades of durability without reintervention—a transcatheter heart valve is expected to undergo SVD over time, necessitating reintervention. For a patient ≤65 years old at index-TAVR, this may mean multiple valve interventions over a lifetime. The previously reported reduction in life expectancy following SAVR has not been observed among TAVR recipients, raising additional concerns regarding the optimal lifetime management strategy for patients with AS [8,30].

### 4.5. Lifetime Management: Coronary Access After TAVR

Preservation of coronary access following TAVR should be addressed. The implantation of a THV within the native or previously replaced aortic valve may obstruct/complicate access to the coronary ostia in the setting of a future acute coronary syndrome, complex coronary artery disease requiring percutaneous intervention, or SVD requiring redo-TAVR [31,32,33,34,35,36]. In younger patients, who may have 20–30 years of expected survival following index-TAVR, the likelihood of requiring coronary intervention at some point is substantial.

### 4.6. Lifetime Management: Redo-TAVR

Valve-in-Valve (ViV) procedures are increasingly being performed with shorter hospital stay and favorable outcomes in both settings (TAV-in-TAV and TAV-in-SAV) as compared to redo SAVR [37,38,39,40,41,42]. However, it presents several challenges. First, THV implantation height at the index-TAVR procedure is a major determinant of the feasibility and safety of future redo-TAVR, with higher implant positions associated with reduced procedural repeatability [13,43,44,45]. THVs are traditionally deployed in a three-cusp and/or cusp-overlap fluoroscopic-projections. With either approach, the true ostium of the left main (LM) coronary artery is not visualized during THV deployment. Implantation depth is an important factor that has been associated with the risk of atrioventricular (AV) conduction disturbance leading to the necessity of permanent pacemaker (PPM) implantation, paravalvular leak or leaflet overhang [46,47]. Conversely, techniques have been developed to reduce implantation depth while reducing conduction disturbances and PPM rates following TAVR. However, higher implants may compromise coronary access and increase the risk of coronary obstruction at the time of TAV-in-TAV procedure [46,48,49,50]. The introduced “Left Main View” implantation technique for visualization of the LM ostium during THV deployment may be of particular value for short-frame THVs, where sub-coronary implantation is possible [51,52]. Used as either a primary or complementary implantation view, this fluoroscopic projection may optimize THV outflow positioning relative to the LM coronary ostium, with potential implications for future coronary access and redo-TAVR feasibility.

### 4.7. Lifetime Management: Index-TAVR and Optimization Strategies

Several procedural strategies have emerged to optimize index-TAVR. Among these, routine post-dilatation at nominal volume (the “double-tap” technique) has been shown to improve THV stent-frame expansion without additional safety concerns [53,54,55,56]. This concept originates from the coronary field, where repeat inflation at the same pressure has been demonstrated to enhance stent expansion [57]. Similarly, combining nominal-volume post-dilatation with complementary fluoroscopic projections, such as the LM-View, may offer incremental benefits in younger patients by supporting a lifetime management approach focused on THV longevity, future coronary access and redo-TAVR feasibility. However, the positive impact on clinical outcomes of these optimization strategies remains speculative.

## 5. Conclusions

The findings from the reviewed studies lead to several key conclusions:

(1) TAVR utilization in patients ≤65 has increased dramatically over the past decade, reaching near-equalization with isolated bioprosthetic SAVR in many North American registries.
(2) There are no randomized controlled trial data comparing TAVR with SAVR specifically in patients ≤65. The low-risk RCTs’ evidence base is derived predominantly from older populations, and extrapolation of these findings to younger patients is not supported by the available evidence.
(3) TAVR recipients ≤65 are a clinically distinct group characterized by significantly heavier comorbidity burdens than SAVR recipients of the same age. They are, in many respects, sicker than both younger SAVR patients and older TAVR recipients on a non-cardiac comorbidity basis. Standard surgical risk models may underestimate the true clinical risk.
(4) Mid-term survival after TAVR in patients ≤65 is significantly worse than after SAVR in matched observational analyses. These findings must be interpreted in the context of the higher comorbidity burden of TAVR recipients, which likely contributes substantially to the observed survival disadvantage.
(5) Substantial hospital-level variation in TAVR utilization exists in patients ≤65, even after risk adjustment, with institutional practice culture and non-clinical factors influencing procedure selection for a significant proportion of patients, including those at the low surgical risk.
(6) Lifetime management considerations (including coronary access preservation, feasibility of redo-TAVR, implantation depth/height strategy, conduction system disturbances, and TAVR optimization techniques such as the double tap method and complementary fluoroscopic views should be incorporated into heart team decision-making when TAVR is considered in young patients.
(7) These findings collectively underscore the need for a randomized controlled trial comparing TAVR with SAVR in patients ≤65 years with severe AS. To enable meaningful comparisons, patients should be considered for randomization only when both TAVR and SAVR are deemed appropriate treatment options by the Heart Team. Until such evidence is available, current ACC/AHA and ESC/EACTS guideline recommendations supporting SAVR as the preferred approach in younger low-risk patients should be upheld, while acknowledging that carefully selected patients with elevated surgical risk or limited life expectancy may derive clinical benefit from TAVR when supported by multidisciplinary heart team evaluation.

## Figures and Tables

**Table 1:** General characteristics of the main studies reporting TAVR in patients aged 65 years and younger.

Study (First Author et al., Year)	Design and Sites	TAVR Patients (n)	Mean/Median Age (yrs)	Male (%)	STS Score/EuroSCORE II	Valve Type and Access	Follow-Up (Months)	Bicuspid Valve Prevalence
Glance et al. 2026 [25]	Retrospective Vizient database (US, 2018–2023)	7765 (<65) among 13,907 AVR patients	59.2 ± 6.2	64.7%	Not reported; claims-based risk	Device type not specified; high adoption of transfemoral access	NR; in-hospital outcomes	0.9% of TAVR vs. 5.4% of SAVR were bicuspid valves
Alabbadi et al. 2025 [23]	State administrative database (California, NY, NJ; 2013–2021)	2708 TAVR (<65) among 9557 AVR patients	61 (IQR 58–63)	63%	Not reported	Device type not reported; transfemoral approach predominated	Up to 6 years (median follow-up); outcomes at 6 years	5.0% of TAVR vs. 14.8% of SAVR before matching; 6.8% vs. 7.5% after matching
Bække et al. 2025 [19]	Multicentre European registry (9 centers; 2010–2019)	459 TAVR patients (≤65)	61 (IQR 57–63)	66%	Median STS 2.6% (IQR 1.4–4.9); 67% low risk, 20% intermediate, 13% high risk	Device type not specified (early-generation balloon/self-expanding); transfemoral approach used in the majority	36 months median follow-up; 3-year outcomes reported	14% bicuspid valves
Gupta et al. 2025 [24]	Regional registry (New England, US; 2015–2023)	274 TAVR patients (<65)	60.8 ± 4.3	61.3%	Median STS 2.5% (IQR 1.2–4.6); only 16.8% considered low risk by heart team	80.6% balloon-expandable, 19.4% self-expanding; 95.3% transfemoral	NR (outcomes reported at 30 days and 1 year)	16.4% bicuspid valves
Ashwat et al. 2025 [21]	Single-center retrospective cohort (US, 2013–2023)	106 TAVR patients (<65)	60.0 ± 4.4	48.4%	Median STS 3.9% vs. 1.8% for SAVR; 52.6% intermediate risk (4–8%), 18.6% high risk (>8%)	68.7% balloon-expandable, 31.3% self-expanding; 86.6% transfemoral	Up to 60 months; 5-year survival reported	24.5% bicuspid valves (unmatched cohort)
Ismayl 2025 [22]	National Readmissions Database (US, 2016–2021)	22,695 TAVR hospitalizations among 75,413 AVR cases aged (<65)	61 (IQR 58–63)	62.5%	Not reported	Device type not reported	30-day and 180-day outcomes	8.8% bicuspid valves vs. 28.1% in SAVR; after matching, 12.6% vs. 11.2%
Coylewright et al. 2025 [20]	Society of Thoracic Surgeons (STS)/American College of Cardiology Transcatheter Valve Therapy (TVT) Registry (US; 2019–2023; balloon-expandable devices only)	13,849 TAVR patients (<65) (5.7% of 139,695)	59.7 ± 4.8	65.5%	Mean STS 3.1% (median 2.0); 39.7% low risk	Balloon-expandable valves; 95.6% transfemoral	Outcomes through 1 year (mortality and readmission)	25.2% bicuspid valves vs. 7.6% in 65–80 y group
Prosperi-Porta 2023 [18]	National administrative database (France, 2015–2020)	1229 TAVR patients (<65) (1.9% of 64,386 TAVR procedures)	NR (young group < 65)	~71%	Charlson comorbidity index stable at 1.3–1.6; no STS data	Device type not reported	Hospital discharge; no long-term follow-up	Bicuspid valve prevalence not reported

Abbreviations: AVR—aortic valve replacement; BAV—bicuspid aortic valve; EuroSCORE II—European System for Cardiac Operative Risk Evaluation II; IQR—interquartile range; NR—not reported; SAVR—surgical aortic valve replacement; STS Score—Society of Thoracic Surgeons Predicted Risk of Mortality; TAVR—transcatheter aortic valve replacement; TVT—Transcatheter Valve Therapy.

## Data Availability

No new data were created or analyzed in this study. Data sharing is not applicable to this article.

## Abbreviations

ACC/AHA	American College of Cardiology/American Heart Association
AS	Aortic stenosis
AVR	Aortic valve replacement
BAV	Bicuspid aortic valve
ESC/EACTS	European Society of Cardiology/European Association for Cardio-Thoracic Surgery
PPM	Permanent pacemaker
RCT	Randomized controlled trial
SAVR	Surgical aortic valve replacement
STS-PROM	Society of Thoracic Surgeons Predicted Risk of Mortality
SVD	Structural valve deterioration
TAV-in-TAV	Transcatheter aortic valve in transcatheter aortic valve
TAVR	Transcatheter aortic valve replacement
THV	Transcatheter heart valve
ViV	Valve-in-valve
